# Factors Affecting Arsenic Methylation in Contaminated Italian Areas

**DOI:** 10.3390/ijerph17145226

**Published:** 2020-07-20

**Authors:** Elisa Bustaffa, Francesca Gorini, Fabrizio Bianchi, Fabrizio Minichilli

**Affiliations:** Unit of Environmental Epidemiology and Disease Registries, Institute of Clinical Physiology, National Research Council, 56123 Pisa, Italy; fgorini@ifc.cnr.it (F.G.); fabriepi@ifc.cnr.it (F.B.); fabrizio.minichilli@ifc.cnr.it (F.M.)

**Keywords:** arsenic pollution, arsenic metabolism, arsenic methylation capacity, human biomonitoring, questionnaire

## Abstract

Chronic arsenic (As) exposure is a critical public health issue. The As metabolism can be influenced by many factors. The objective of this study is to verify if these factors influence As metabolism in four Italian areas affected by As pollution. Descriptive analyses were conducted on 271 subjects aged 20–49 in order to assess the effect of each factor considered on As methylation. Percentages of metabolites of As in urine, primary and secondary methylation indexes were calculated as indicators for metabolic capacity. The results indicate that women have a better methylation capacity (MC) than men, and drinking As-contaminated water from public aqueducts is associated with poorer MC, especially in areas with natural As pollution. In areas with anthropogenic As pollution occupational exposure is associated with a higher MC while smoking with a poorer MC. Dietary habits and genetic characteristics are probably implicated in As metabolism. BMI, alcohol consumption and polymorphism of the AS3MT gene seem not to influence As MC. Arsenic metabolism may be affected by various factors and in order to achieve a comprehensive risk assessment of As-associated disease, it is crucial to understand how these factors contribute to differences in As metabolism.

## 1. Introduction

Arsenic (As) is a toxic metalloid ubiquitously distributed into the environment. The World Health Organization (WHO) and the International Agency for Research on Cancer (IARC) classified As and its compounds as human carcinogens [1,2]. In fact, it is well established that As chronic exposure is associated with both carcinogenic and non-carcinogenic effects in a dose-responsive pattern [2,3,4]. Humans are exposed to As principally through contaminated water (for consumption, cooking or irrigation) and food, industrial processes and smoking tobacco [5]. The main As-contributors in the human diet are fish, shellfish, meat, milk and cereals although food As exposure is generally lower than water As exposure [6,7]. Environmental exposure to inorganic arsenic (iAs), especially from drinking water or occupational settings, has caused calamity in several countries [8,9,10,11].

As a result of the toxic effects of As on human health, many nations have adopted regulatory standards to limit the amount of As in drinking water. In Italy, the Directive 98/83/CE [12], in force since 2003, imposed the limit value of 10 µg/L for As in drinking water. Considered the Italian specific geological conditions determining the natural occurrence of As in the aquifers used for drinking water production, Italy asked and obtained, in four regions, two derogations to 50 µg/L (for the period 2003–2009), and a third one at 20 µg/L (for the period 2010–2012) [13]. Since the Italian great concern about the health risk to exposure to low-to-moderate As concentrations in drinking water [7,14,15], an epidemiological study called SEpiAs (Epidemiological Surveillance in areas with natural or anthropic Arsenic pollution, funded by the Italian Ministry of Health) was carried out in four As-contaminated areas. The aim of SEpiAs was to assess the relationship between human As exposure and biological markers of early health effects, in order to define indicators for an advanced environmental public health surveillance.

To date, it is unclear whether there are subjects more susceptible to the toxic effects of As and not adequately protected by the current drinking water standard. Differences in As metabolism or in the prevalence of As-associated diseases among people of either gender, various age groups, or different ethnic groups might be indicators of differences in susceptibility to As toxicity [16]. A crucial factor affecting the scientific understanding of As toxicity, which has limited epidemiological research on possible variations in disease susceptibility, is the complexity of As metabolism [17]. In fact, for As metabolism currently exists a generally accepted pathway and a newly one proposed pathway. In the generally accepted pathway, after exposure, biotransformation of As takes place in the body and the inorganic trivalent arsenite is a preferential substrate for As (+3 oxidation state) methyltransferase (AS3MT). The As metabolism consists in an alternation of reduction and oxidation reactions in which the pentavalent As species are formed before the respective trivalent species, and mono- and dimethylated metabolites are generated sequentially [18,19]. The reduction of pentavalent monomethylarsonic acid (MMAV) to monomethylarsonous acid (MMAIII) may be the rate-limiting step in the metabolism of iAs [20,21]. As the methylation process is incomplete, iAs along with MMA and dimetilarsinic acid (DMA) are excreted in the urine and the relative proportion of urinary arsenic (uAs) species is considered an index of an individual’s As methylation capacity (MC). Therefore, As toxicity is strictly related to its metabolism, which in turn is highly dependent on the methylation status and valence of its metabolites [22,23].

The newly proposed pathway suggests that trivalent methylated As species might be formed before the respective end-products of pentavalent species [24]. This pathway is compatible with the concept that “oxidation is detoxification of arsenic” [25], because trivalent methylated As species are suggested to be more toxic than the pentavalent methylated arsenic species and the organic species [26,27,28,29]. Nonetheless, this hypothetical pathway requires further confirmation.

Although the uAs profile in various population is generally 10–30% iAs, 10–20% MMAV, and 60–80% DMAV [30], there are however substantial variations in As metabolism at individual and population levels [16,31] that could be one of the possible explanation for the inter-individual susceptibility to As-induced health hazards [32]. In case of long-term exposure to As from drinking water, uAs concentration could be a better marker than in drinking water or hair [31]. Among As-exposed subjects, an increased urinary MMAV suggests an inefficient methylation and probably an increased concentration of the highly toxic MMAIII at cellular level. Previous epidemiological studies showed that a higher proportion of urinary MMA (MMA%) and a lower proportion of urinary DMA (DMA%), which is believed to indicate a less efficient iAs metabolism, are associated with an increased risk of skin lesions [33,34,35,36], cancer [37,38,39,40,41,42,43,44], peripheral vascular disease [11,45,46,47], hypertension [45], and oxidative stress status [48]. Nevertheless, in contrast to most iAs-associated health outcomes, lower MMA% and higher DMA% that are believed to indicate a more efficient As metabolism, are associated with a more elevated risk of cardiometabolic disorders, including diabetes [49,50,51,52]. Therefore, As toxicity is closely related to its metabolism and is highly dependent on the methylation status and the valence state of the metabolites.

Growing interest has recently focused on the factors influencing As methylation. Besides the As exposure, individual’s ethnicity [17], age, gender, Body Mass Index (BMI) [34,47,53], tobacco smoking [54], dietary habits [55,56], and inherited genetic characteristics [57,58,59,60,61] were reported to be related to MC.

The objective of this article is to verify whether and how the factors listed above are able to modify the As metabolism in SEpiAS subjects and eventually identify other determinants responsible for inter-individual variation of As MC.

## 2. Materials and Methods 

A summary of the study areas, samples, data collection, urine sample collection, As analyses and genetic susceptibility is following. All the detailed information on the SEpiAs materials and methods were previously published [15].

### 2.1. Study Areas

SEpiAs was carried out in four Italian areas, two mountainous/hilly areas located in central Italy (Amiata in Tuscany, and Viterbese in Latium where As was of a natural origin, with contamination of soil and water) and in two municipal areas in southern Italy (Taranto in Apulia and Gela in Sicily where As was anthropogenic, due to the contamination of soil, water and air associated with industrial activities). The industrial areas of Taranto and Gela were declared by the Ministry of Environment as National Priority Contaminated Sites on the basis of documented environmental contamination and/or presence of hazardous waste [7,62,63,64].

### 2.2. Study Sample

SEpiAs was designed as an epidemiological sample survey based on Human BioMonitoring (HBM). Out of 500 residents, randomly selected from the municipal registries and stratified by area, gender, and age (20–29, 30–39, 40–44 years), 341 subjects were contacted and invited to participate in the study. For each area and gender, the percentages of subjects sampled by the three age classes were 40%, 40%, and 20%, respectively. A total of 271 subjects were recruited between 2011 and 2013 (participation rate: 79.4% of the 341 contacted subjects, and 93.4% out of the 290, which is the final sample) (Table 1). A urine specimen from these 271 subjects was also collected. Each subject was told not to consume fish for three days before the urine collection. This information was also checked by both the questionnaire and the organic uAs level.

All subjects gave their informed consent for inclusion before their participation in the study. The study was conducted in accordance with the Declaration of Helsinki, and the protocol was approved by the Ethics Committee of the Local Health Units of Viterbo, Caltanissetta (for Gela), Siena (for Amiata) and Taranto. Project Identification Code: B51J10001120005.

### 2.3. Urine Sample Collection and Arsenic Analysis

uiAs, MMA and DMA were measured using a dynamic reaction cell inductively coupled plasma mass spectrometer, after chromatographic separation with high-performance liquid chromatography. The method limits of quantification for the different As species were in the range of 0.1–0.2 µg/L. Quantification was carried out by a seven point matrix-matched calibration in the range 0.1–20 µg/L. The accuracy for the determination of total As was tested by analyzing the quality control material Lyphochek 1 (urine metal control level 1, Bio-Rad, Irvine, CA, USA). The target value was 71 µg/L. The average concentration from day to day (*n* = 20) was 70 µg/L (RSD = 6.5%), a value in a very good agreement with the target value for total As [65]. As to MMA, DMA, AsIII, and AsV, urine samples spiked with 20 µg/L of each As species, were analyzed (day-to-day, *n* = 20), and the average recovery was between 90% and 105%. Average intra- and inter-day repeatability, determined for total As and each As species, was <5%. Concentrations below the limit of detection (LOD) of 0.2 µg/L, due to the instrument’s inability to detect extremely low levels of chemicals, were found in less than 10% of sampled subjects; a value of 0.141 (LOD/$\sqrt{2}$) was assigned to measurements that were less than the LOD [66].

### 2.4. Genetic Susceptibility

In order to define different metabolic and reparative capacities, the presence of specific functional polymorphisms of genes involved in metabolic detoxification mechanisms was assessed. A genetic susceptibility assessment was performed by evaluating a set of polymorphisms considered to be associated with As methylation, such as AS3MT Met287Thr polymorphism in the AS3MT gene and glutathione S-transferase polymorphisms (GST-T1) [67,68,69].

### 2.5. Urine Arsenic Indicators

Concentrations of uiAs, MMA, DMA and total As (TAs) were used as urine As indicators. The percentages of uiAs (iAs%), MMA (MMA%) and DMA (DMA%) were defined as iAs/TAs×100%, MMA/TAs×100% and DMA/TAs×100%, respectively. Two methylation indices, the Primary Methylation Index (PMI) [(MMA+DMA)/TAs or MMA/iAs] and the Secondary Methylation Index (SMI) [DMA/(MMA+DMA) or DMA/MMA] were calculated to assess As MC.

### 2.6. Data Collection and Questionnaire Variables Selection

For the 271 subjects recruited, individual data on residential history, socio-economic status, environmental and occupational exposures, lifestyle, and dietary habits, were collected through a questionnaire.

Variables considered in the literature associated with As methylation were selected, namely gender, smoking habits, alcohol consumption and BMI [23]. We also included some variables that emerged as informative in a recent study performed using SEpiAs data [70], i.e., the area of residence, water consumption, exposure to chemical and physical agents, dietary habits and some gene polymorphisms (Table 2). Particularly, the variables for which more than two exposure classes were defined in the questionnaire were dichotomized as described in the “Value” column in Table 2 (exposure: yes/no, consumption: yes/no).

### 2.7. Statistical Methods

Bivariate analyses were performed on 271 subjects, to assess the effect of each factor considered (variables in Table 2) on As methylation in term of standardized mean differences (SMDs) with confidence intervals at 95% of probability (95% CI). The differences between the means were tested by t-test and all the *p*-values were reported (Table 3).

Subsequently, bivariate analyses were carried out for each factor coupling the areas by the source of As pollution. In particular, the areas were coupled as follows: Amiata+Viterbese (AV; 130 subjects) for As pollution of natural origin and Taranto+Gela (TG; 137 subjects) for anthropogenic As pollution. Additionally, in this case the results are reported as SMDs with 95% CI and a two-sided *p*-value (Table 3).

Consistently with the recent scientific literature on the “non-use” of the statistical significance threshold for defining the results to be discussed [71], we commented both those that actually are accompanied by a statistical significance but also those that, in our opinion, can provide interesting ideas to discuss or to deepen with further studies. 

## 3. Results

### 3.1. Effects of Gender on Arsenic Methylation

Compared to women, men of the TG area had a higher MMA%, a higher PMI and a lower SMI (Table 3). These results were also observed on the total of the four study areas (Table 3).

### 3.2. Effects of Age on Arsenic Methylation

While considering the four areas as a whole, results did not show any interesting signals (Table 3), from analyses on coupled areas statistically significant results among AV subjects and some signals among TG subjects were observed (Table 3). Particularly, the AV subjects aged 30-39, compared to those aged 20–29, had a higher iAs%, a lower DMA% and a lower SMI (Table 3). TG subjects aged 30–39, compared to those aged 20–29, showed a higher SMI, highlighting an opposite situation respect to that observed among AV subjects (Table 3).

### 3.3. Effects of Aqueduct Water Consumption for Drinking Purpose on Arsenic Methylation

In the AV area, subjects drinking aqueduct water had a higher MMA%, a lower DMA%, a higher PMI and lower SMI compared to those who did not (Table 3). Results for the TG area showed the same trend but were not statistically significant. These results were also observed on the total of the four study areas (Table 3).

### 3.4. Effects of Occupational Exposure to Chemicals on Arsenic Methylation

Subjects of the TG area occupationally exposed to chemicals showed a lower MMA% and a higher DMA%, a lower PMI and a higher SMI compared to those not exposed (Table 3). These results were confirmed considering all the four study areas (Table 3).

### 3.5. Effects of Smoking 0n Arsenic Methylation

Among TG subjects who still smoke or have smoked, with an increase of one pack-year of cigarettes a lower DMA% and a lower SMI were observed (Table 3). These results were also observed on the total of the four study areas (Table 3).

### 3.6. Effects of Wine Consumption on Arsenic Methylation

Compared to those who did not drink wine, individuals of the AV area drinking at least 1–2 glasses of wine per day had a higher iAs%, a lower DMA% and a lower SMI (Table 3). The TG area showed the same trend even if not statistically significant. The observed results for iAs% and DMA% were confirmed considering all the four study areas (Table 3).

### 3.7. Effects of Whole Milk Consumption on Arsenic Methylation

Compared to those who did not drink whole milk, among subjects of the AV area who consumed at least 1–2 glasses of whole milk per week a lower MMA% and a lower PMI were observed (Table 3). Even if signals were weaker, the TG area showed similar results with a lower MMA%, a lower PMI, in addition to a higher iAs% (Table 3). Analyses performed on the four study areas provided the same results (Table 3).

### 3.8. Effects of Meat Consumption on Arsenic Methylation

Subjects residing in TG area and consuming 4 different types of meat at least 1–2 times a week had a lower MMA% and a lower PMI in comparison to those who did not (Table 3). Considering the four areas as a whole, any significant finding was detected (Table 3).

### 3.9. Effects of Fish Consumption on Arsenic Methylation

Respect to those who did not eat fish, subjects who consumed fish, mollusks and crustaceans at least 1-2 times a week showed a higher iAs% (Table 3).

The higher iAs% observed in all the four areas was due to the AV area. Moreover, among AV area subjects, a lower DMA% and a lower SMI were observed. Conversely, a lower MMA% and a lower PMI were detected among the TG subjects but not among those residing in the AV area (Table 3).

### 3.10. Effects of the Polymorphism of the As3mt Gene on Arsenic Methylation

While considering the four areas as a whole, results did not show any interesting signal (Table 3), coupling the areas, AV subjects showing the mutated allele of AS3MT gene, had a higher SMI respect to those who did not show the mutated allele (Table 3).

### 3.11. Effects of The Polymorphism of The Gstt Gene on Arsenic Methylation

Compared to the individuals who did not carry the wildtype of the GSTT1 gene, the wildtype carriers showed a lower iAs% and a higher MMA% (Table 3).

These results seemed to be partially due to the TG area. In this area the values of the urinary profile, although slightly different, have the same trend as those of the total areas. In fact, the previous significant lower iAs% was not detected while was still observed the higher MMA% among the wild type carriers of the GSTT gene (Table 3). In these subjects a higher PMI was further reported (Table 3).

Finally, we did not find any statistically significant results when considering BMI, alcohol consumption and some kind of foodstuff, such as pasta and cereals.

### 3.12. Summary of The Results

Considering gender, the signals collected in the analyses on the four areas were only attributable to the TG area with anthropogenic As pollution. As regards the aqueduct water consumption, the signals observed in the four areas seem to be due to the AV area with natural As pollution, while in the case of occupational exposure to chemicals, anthropogenic As pollution of TG area represented the major contribution. As for dietary habits, results observed in the analyses performed on the four areas were due to the AV area in the case of wine and fish consumption, while for whole milk consumption were due to both the AV and TG areas. The results on the GSTT1 gene polymorphism seem to be related to the TG area.

Notably, some factors were not significant in the four areas as a whole but were instead significant when considering the coupled areas. For example, statistically significant results were observed among the subjects of the AV area for three methylation indexes and among the subjects of the TG area for the SMI. Even in the case of meat consumption, the analyses on the four areas did not report any statistically significant findings but statistically significant results on coupled areas among the TG subjects. Similar observations can be applied in the case of fish consumption, for which statistically significant results were detected for both the AV and TG areas, observing a worse methylation among AV subjects and a better methylation among TG subjects.

## 4. Discussion

The aim of this paper was to verify whether the factors suggested by the scientific literature can influence and how the As MC of the studied population. We also set out to identify any other factor that could affect this ability. The results indicate that women have a better MC than man, and both drinking water from public aqueducts and wine consumption are associated with a poorer MC, particularly in areas characterized by natural As pollution. In areas with anthropogenic As pollution occupational exposure is associated with a higher MC while smoking with a poor As MC. Dietary habits provide inconsistent results but provide signs of possible implications in As metabolism, which will need to be further elucidated. On the other hand, BMI, alcohol consumption and polymorphism of the AS3MT gene do not appear to be factors influencing As MC in the studied population.

Arsenic exposure has been shown to increase the risk of certain diseases, both carcinogenic and non, such as As-induced skin lesions [72,73], peripheral artery disease [74], hypertension [75,76], cardiovascular disease [77], diabetes [78], skin and urothelial carcinoma [79]. Even if the average urinary values for iAs%, MMA% and DMA% among As-exposed population are 10–30%, 10–20% and 60–70%, it has been suggested that a specific methylation profile is associated with increased incidence of As-related diseases [11,36,45].

**Figure 1:**
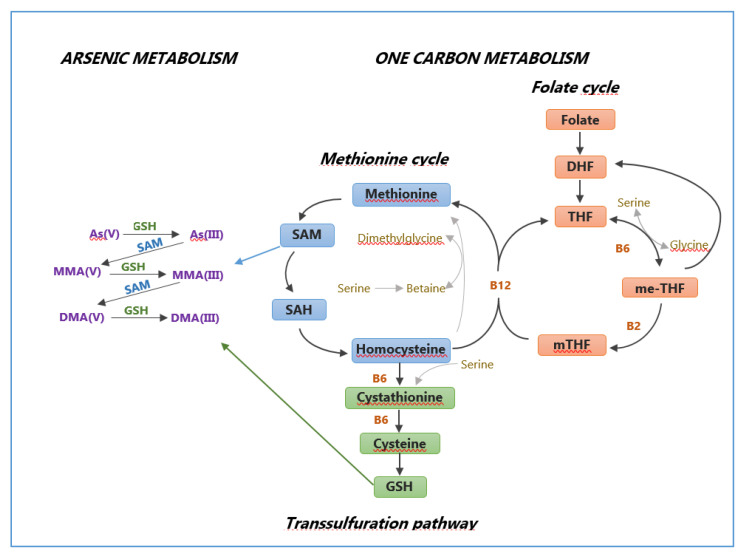
Interaction between one carbon metabolism and arsenic metabolism. Legend—As: arsenic; B2: vitamin B2 (riboflavin); vitaminB6: vitamin B6 (pyridoxine); B12: vitamin B12 (Cobalamin); DHF: dihydrofolate; DMA: dimethyl arsenic; GSH: glutathione; me-THF: 5,10-methylenetetrahydrofolate; MMA: monomethyl arsenic; mTHF: 5-methyltetrahydrofolate; SAH: s adenosylhomocysteine; SAM: s adenosylmethionine; THF: tetrahydrofolate. Notes—In one carbon metabolism (OCM), a carbon unit from serine or glycine is transferred to THF to form me-THF which in turn is reduced to m-THF which used to methylate homocysteine to form methionine catalyzed by a B12-containing methyltransferase. Much of the methionine which is formed is converted to SAM which is also the main donor of methyl groups. The As biotransformation involves the imbalance of the OCM, and this mainly occurs through three ways: 1. During the first step of As biotransformation SAM is required but it is also the main donor of methyl groups for the DNA methyltransferases necessary for DNA methylation, which therefore is missing; 2. The synthesis of SAM requires homocysteine (Hcy) which is usually used to create GSH which has an antioxidant action. If a lot of Hcy is required to form SAM which gives methyl groups for the As biotransformation, it does not form GSH and therefore there is no antioxidant action; 3. Dietary folates can enter OCM as 5-methyl-THF, which is involved in the formation of DNA and RNA precursors but which can donate a methyl group to Hcy to form SAM. Since a lot of SAM is required for As methylation, 5-methyl-THF does not perform its function because it gives methyl groups for the synthesis of SAM.

A better MC in women than in men found in the four study areas is in agreement women with most of the evidence in the literature [23,34,80,81,82,83,84]. Estrogen, choline, betaine, vitamin B12, folate and As methylation are linked by precise sex-linked metabolic pathways (Figure 1). 

Particularly, estrogen up-regulates phosphatidylethanolamine-N-methyltransferase leading to de novo synthesis of choline. Choline and folate both in turn regulate the synthesis of S-adenosyl methionine (SAM), which is the methyl donor for As methylation [85,86]. Thus, even if indirectly, estrogen may promote As metabolism. It is also reasonable to observe a better As methylation in women before menopause than in age-comparable men, and probably a similar or even poorer As methylation in women after menopause. Furthermore, men can be exposed to a higher number of factors that inhibit As methylation, such as drinking or smoking.

While the results of the analyses by age classes on the four areas did not report any statistically significant results, TG area subjects aged 30–39 exhibited a worse As MC (higher MMA% and lower DMA% and SMI), than those aged 20–29 accordingly with what reported by Huang et al. [81] and these conditions are known to be potential risk factors for arsenicosis [87]. It is worth of note that our age classes are quite different from those usually defined (>50 vs. ≤50 years). Generally, there is inconsistent evidence about the age effect on As metabolism due to the lack of adjustment for potential confounders, since aging can be associated with a variety of changes in the organs. In fact, the senescence of many organs may block the As methylation process and older age can be associated with longer As exposure. Hence, older people could have a poorer MC and a major susceptibility to As damage [23]. 

In our study, among those who drink water from public aqueducts in the four areas, statistically significant higher MMA%, lower DMA%, a higher PMI and a lower SMI were observed, indicating an inefficient As methylation. These results were principally due to the AV subjects for whom drinking water from public aqueduct was the main route of As exposure. Our results were in agreement with a recent meta-analysis suggesting that the As MC declines when doses of exposure increased [23]. By this way chronic As exposure may lead to insufficient metabolic-associated factors or enzymes such as SAM and GST (see the specific paragraph on dietary habits). Inefficient As methylation has been also observed for low As exposure (<50 µg/L) [23] and this range of As concentrations was similar to the concentrations of some parts of the AV area.

In all the four study areas, subjects occupationally exposed to chemicals showed a higher As MC compared to those who were not (higher, but not statistically significant iAs%, higher DMA% and SMI), although exclusively in the TG area. To date, most occupational studies relating As exposure to urinary excretion have been conducted in non-ferrous smelters reporting a decrease in As MC (higher iAs% and MMA% and lower DMA% and PMI) [88,89]. The decrease in As MC may be caused by the methyltransferase inhibition or the depletion of reduced glutathione (GSH) required for As methylation, which led to either iAs saturation or inhibition of methylation in copper smelter workers. A study on workers from a steel and iron smelting plant showed instead a significant association between higher TAs urinary levels and increased SMI, represented by low iAs% and high DMA% [90]. Furthermore, although several studies suggested that some types of seafood may contain DMA, which can interfere with specific contribution of urinary As species after iAs exposure [91,92,93], studies on workers exposed to As observed that urinary As species were not influenced by the presence of dietary organoarsenicals [88,90]. Our results are consistent with those on steel workers but not with results from non–ferrous smelter workers. Additionally, in our study, DMA concentration in urine is not influenced by seafood assumption, since subjects were asked not to eat fish 3 days before the urine collection. There are probably other factors influencing the urinary As profile and the As MC in subjects occupationally exposed to chemicals or exposed to organic solvents that need to be analyzed in further studies.

An association between smoking and a poorer As MC has been speculated, with smokers having a higher MMA% and lower both DMA% and SMI than non–smokers [11,81,82,94]. Our data confirm these findings observing lower DMA% and SMI with the increment of one pack–year of cigarettes, particularly in the areas with anthropogenic As pollution. It is known that both As exposure and tobacco smoking can promote oxidative stress and tissue damage stimulating cells to release free radicals and consume antioxidants [22]. In addition, several enzymes or co–factors involved in the second methylation phase can be negatively affected by chemicals contained in cigarettes. Cigarettes may also contain As, making smoking a further pathway for As exposure, but the As content in cigarettes is so small that the exposure dosage from smoking is often negligible if compared to other exposure routes such as contaminated drinking water [23]. As previously noted, it is possible that some confounding factors exist, particularly male gender that could partly contribute to a poorer MC associated with smoking, since a much higher proportion of men than women are smokers.

Although our results on alcohol consumption are not statistically significant, subjects drinking at least 1–2 glasses of alcohol per day had a urinary As profile indicating a poorer MC with higher iAs%, MMA% and lower DMA%, as also reported by Hopenhayn–Rich et al. [95]. Instead, our results are statistically significant among subjects of all the four areas drinking at least 1–2 glasses of wine per day compared to non–drinkers indicating a poorer MC (higher iAs%, lower DMA% and lower SMI). These same findings were observed among the AV subjects who also presented a lower SMI. Among TG subjects, associations were similar but not statistically significant. Overall, these results are in accordance with prior studies reporting that an elevated daily alcohol consumption can negatively affect uAs concentration [96,97]. Moreover, As methylation processes could be affected by alcohol (wine) intake as a consequence to damage to the liver, the main organ associated with As metabolism [23].

As regards dietary habits, in all the areas, particularly in the AV area, subjects consuming at least 1–2 glasses of whole milk per week had a lower MMA% and PMI, indicating that milk consumption could enhance As methylation. Moreover, the meat consumption for at least 1–2 times a week seemed to positively influence the As MC only among the TG area subjects. On the other hand, for those who consume fish at least 1–2 times a week, compared to non–consumers, the analyses on the four areas showed only an increase in iAs%, while the AV subjects had a scarce MC (higher iAs%, lower DMA% and SMI) and the TG subjects showed lower MMA% and PMI, which are signs of an efficient MC. Nonetheless, it should be noted that most of the studies evaluating the capability of the dietary habits to influence As metabolism were not conducted considering the type of food but rather those micronutrients known to influence As metabolism. In contrast, our study did not assess the real nutritional status of the subjects since a food frequency questionnaire would be needed to know the amount of all those substances capable to modify both metabolism and toxicity of As (e.g., folate, vitamin B12 (cobalamin), betaine, choline, cysteine and methionine) [22,98]. The As methylation reactions depend on SAM, the main donor of methyl groups [99] and the SAM availability also depends on nutritional factors (folate, creatinine vitamin B12, choline, betaine) involved in One–Carbon Metabolism (OCM), a biochemical pathway responsible for the DNA synthesis and repair and also for synthesis of both SAM and GSH (Figure 1) [100]. In fact, folate intake and urinary creatinine (a strong predictor of As MC) are negatively associated with iAs% and positively associated with DMA% [98]. Vitamin B12 has been inconsistently linked to As metabolism in human studies reporting positive association with iAs% and negative association with DMA% [56], null results [101] or contrasting directions of association [102]. Dietary choline intake is positively associated with the SMI, inversely associated with MMA% and positively associated with DMA% [102,103] and dietary betaine has a weaker association with As methylation than choline [102,103]. Nonetheless, our analyses on the dietary habits of the studied population, although did not evaluate the intake of micronutrients, showed that the nutritional status had a likely role in regulating As methylation and thus in As–related health hazards to humans according to the current growing scientific evidence [22,98].

There is a large variation in susceptibility to toxic effect by iAs among individuals and ethnics, depending on the difference in As metabolism [16]. Polymorphism(s) can occur in the genes for AS3MT and GST that are responsible for the metabolism of As compounds and therefore may contribute to the variability in iAs biotransformation [16,17,19,104]. Although both of these enzymes can influence As metabolism and then the concentration of As metabolites in the urine, in our study, statistically significant effects were observed exclusively for polymorphism of the GSTT gene. In the four areas, a decreased iAs% and an increased MMA% were found among carriers of GSTT1 wild type compared to those with the GSTT1 null polymorphism, and in particular the TG subjects exhibited an increase in both MMA% and PMI. Polymorphisms in GST genes may have effects on the behavior of several enzymes involved with the maintenance of cellular GSH levels [105]. Phase II metabolic GST enzymes conjugate metabolic intermediates into more soluble forms that are subsequently excreted. It may be hypothesized that when an individual lacks the enzyme activity (null polymorphism) and is exposed to a xenobiotic compound, he would be at a greater risk of disease [105,106]. Conversely, subjects with high detoxifying ability may be at greater risk of adverse effects associated with chronic As exposure through drinking water, since the products of primary and secondary methylation (MMAIII and DMAIII) are suspected to be more reactive than their metabolic precursors (MMAV and DMAV) [27,107]. Epidemiological studies reported contradictory results. GSTT1 wild/null polymorphism could have no relevance to urinary As in the populations studied [108,109]. while in other studies individuals who were GSTT1 null had an elevated DMA% in urine [110], a higher proportion of MMA, a significantly higher PMI and a significantly lower SMI [60,111]. Our results do not confirm previous findings and although GSTT1 could be involved in As metabolism and influence the internal dose of methylated As, its role remains to be fully elucidated.

Epidemiological studies evaluating the relationship between BMI and the distribution of urinary As metabolites found a positive association of BMI with DMA% and a negative association with MMA% [112,113,114,115,116]. Conversely, our results did not show any association between BMI and urinary As metabolite levels, a findings that is instead in accordance with what reported by Bae et al. [95]. This discrepancy is possibly attributable due to the lack of data on the BMI that allowed us to carry out analyses on approximately 67% of the total sample.

## 5. Conclusions

In summary, the As methylation pattern can be influenced by a variety of environmental factors such as age, gender, As exposure level, smoking habits, nutritional status and dietary factors. Gender, As–exposure through consumption of drinking water, occupational exposure and dietary habits have a certain impact on As methylation. The changes observed in As methylation may also depend on the source of As exposure that is natural or anthropogenic. Moreover, to date susceptibility to As–induced health hazards has been acknowledged to differ among individuals and this could be partially ascribed to inter–individual differences in As metabolism. We trust that our results will help in understanding the As methylation pathway and the factors affecting its metabolism which are recognized to be of fundamental importance for the risk assessment of iAs–associated diseases.

## Figures and Tables

**Table 1:** Distribution by area, gender and age (20–29, 30–39, 40–44 years) of the 271 subjects recruited after the consent to the interview and the collection of a urine sample. The percentage in brackets was calculated on the contacted subjects by area. For example: Amiata area, age class 30–39, 12 males (75%). It means that on 16 subject contacted, only 12 were recruited, i.e. 75% of those contacted.

AREA	MALE				FEMALE				TOTAL			
	20–29 (%)	30–39 (%)	40–44 (%)	TOTAL (%)	20–29 (%)	30–39 (%)	40–44 (%)	Total (%)	20–29 (%)	30–39 (%)	40–44 (%)	Total (%)
Amiata	10 (62.5)	12 (75.0)	6 (75.0)	28 (70.0)	11 (68.7)	11 (68.7)	8 (100.0)	30 (75.0)	21 (65.6)	23 (71.9)	14 (87.5)	58 (72.5)
Viterbese	15 (88.2)	11 (68.8)	6 (66.7)	32 (76.2)	16 (94.1)	15 (93.8)	9 (100.0)	40 (95.2)	31 (91.2)	26 (81.3)	15 (83.3)	72 (85.7)
Taranto	11 (84.6)	9 (69.2)	4 (66.7)	24 (75.0)	11 (84.6)	10 (76.9)	5 (83.3)	26 (81.3)	22 (84.6)	19 (73.1)	9 (75.0)	50 (78.1)
Gela	16 (69.6)	20 (86.9)	12 (100.0)	48 (82.8)	23 (100.0)	12 (52.2)	8 (66.7)	43 (74.1)	39 (84.8)	32 (69.6)	20 (83.3)	91 (78.4)
TOTAL	52 (75.4)	52 (76.5)	28 (80.0)	132 (75.9)	61 (88.4)	48 (70.6)	30 (85.7)	139 (80.8)	113 (81.9)	100 (73.5)	58 (82.6)	271 (78.8)

**Table 2:** Questionnaire variables selected for the statistical analyses.

Description	Value
Personal data	
“Is the subject female?”	Dichotomous
Age class	20–29, 30–39, 40–44 (years)
Residence area	Amiata, Viterbo, Taranto, Gela
“Has the subject a body mass index>25?”	Dichotomous
Water	
“The subjects habitually drinks water from public aqueduct”	Dichotomous
Exposure to chemical and physical agents	
“Has the subject ever worked in a chemical industry?”	Dichotomous
Smoking	
Lifetime tobacco exposure calculated as pack/year=(number of cigarettes smoked per day*number of years the person has smoked)/20	Continuous
Alcohol consumption	
“Does the subject consume at least 1–2 glasses of wine a day?”	Dichotomous
“Does the subject consume at least 1–2 glasses of any kind of alcoholic beverages * a day?” *(beer/super alcoholic beverages/aperitifs or mixture of non-alcoholic and super alcoholic beverages/grappa/whiskey/cognac and similar)	Dichotomous
Dietary habits	
“Does the subject consume at least 1–2 times a week whole milk?”	Dichotomous
“Does the subject consume at least once or twice a week these types of meat: poultry, beef, pork, lamb, horse, pork products, liver ?”	Dichotomous
“Does the subject consume at least 1–2 times a week fish, mollusks or crustaceans?”	Dichotomous
”Does the subject consume at least 1–2 times a week cereals, pasta and bread?”	Dichotomous
“Does the subject consume wine at least 1–2 times a week?”	Dichotomous
Genetic polymorphisms	
“The subject has at least one mutated allele of the As (+3 oxidation state) methyltransferase gene”	Dichotomous
“The subject carries the wild type genotype of the glutathione S-transferase gene”	Dichotomous

**Table 3:** Effects of factors analyzed on arsenic methylation considering the 4 areas as a whole and areas coupled by the origin of arsenic pollution, i.e. natural arsenic pollution (AV area) and anthropogenic arsenic pollution (TG area). Results are reported as Standardized Mean Differences (SMDs) with the 95% Confidence Interval (95%CI) and the *p*-value for the arsenic methylation indexes, including the percentage of inorganic arsenic (iAs%), percentage of monomethyl arsenic (MMA%), percentage of dimethyl arsenic (DMA%), Primary Methylation Index (PMI) and Secondary Methylation Index (SMI).

**Gender–Men vs. Women**											
		**iAs%**	***p***	**MMA%**	***p***	**DMA%**	***p***	**PMI**	***p***	**SMI**	***p***
AV area	SMD [95%CI]	−0.64 [−4.90–3.63]	0.767	0.47 [−3.14–4.09]	0.434	1.72 [−2.31–5.74]	0.400	0.02 [−0.28–0.32]	0.904	0.14 [−0.25–0.53]	0.482
TG area		−2.25 [−7.03–2.53]	0.353	**5.64 [2.54–8.74]**	<0.001	−3.39 [−7.84–1.06]	0.134	**0.39 [0.16–0.67]**	0.001	**−1.39 [−2.62–(–0.17)]**	0.026
Total of 4 areas		−1.62 [−4.82–1.59]	0.321	**2.48 [0.38–4.59]**	0.021	−0.87 [−3.87–2.13]	0.570	**0.22 [0.03–0.41]**	0.026	**−0.70 [−1.38–(−0.03)]**	0.040
**Age class—class 2 (30–39) vs. class 1 (20–29) (1st row) and class 3(40–49) vs. class 1 (20–29) (2nd row)**											
		**iAs%**	***p***	**MMA%**	***p***	**DMA%**	***p***	**PMI**	***p***	**SMI**	***p***
AV area	SMD [95%CI]	**4.44 [−0.28–9.16]** −2.61 [−8.11–2.88]	0.065 0.349	1.10 [−1.98–4.17] 1.90 [−1.68–5.48]	0.481 0.297	**−5.54 [−9.98–(–1.09)]** 0.72 [−4.46–5.89]	0.015 0.785	−0.20 [−0.54–0.15] 0.14 [−0.26–0.54]	0.258 0.483	**−0.54 [0.98–(−0.11)]** −0.14 [−0.97–0.36]	0.014 0.570
TG area		0.92 [−4.55–6.40] −0.19 [−6.49–6.11]	0.739 0.952	−2.62 [−6.31–1.06] −0.83 [−5.07–3.41]	0.161 0.699	1.70 [−3.42–6.82] 1.02 [−4.87–6.92]	0.513 0.732	−0.10 [−0.37–0.17] −0.14 [−0.45–0.18]	0.466 0.390	**1.45 [0.041–2.86]** 0.77 [−0.85–2.39]	0.044 0.346
Total of 4 areas		2.61 [−1.02–6.24] −1.37 [−5.57–2.83]	0.158 0.522	−0.78 [−3.19–1.64] 0.49 [−2.31–3.28]	0.527 0.731	−1.83 [−5.24–1.57] 0.88 [−3.06–4.82]	0.290 0.660	−0.15 [−0.36–0.07] −0.00 [−0.25–0.25]	0.187 0.985	0.46 [−0.31–1.23] 0.33 [−0.56–1.22]	0.240 0.466
**Body Mass Index—Overweight (≥25) vs. Normal Weight (<25)**											
		**iAs%**	***p***	**MMA%**	***p***	**DMA%**	***p***	**PMI**	***p***	**SMI**	***p***
AV area	SMD [95%CI]	−0.62 [−1.77–0.52]	0.279	0.60 [−0.36–1.56]	0.213	0.02 [−1.19–1.24]	0.970	−0.041 [−0.17–0.08]	0.510	−0.33 [−0.14–0.07]	0.540
TG area		−0.07 [−0.50–0.36]	0.739	0.068 [−0.22–0.36]	0.645	0.00 [−0.40–0.40]	0.982	0.00 [−0.02–0.021]	0.982	0.00 [−0.12–0.11]	0.949
Total of 4 areas		−0.05 [−0.43–0.34]	0.814	0.00 [−0.27–0.29]	0.959	0.04 [−0.33–0.40]	0.835	−0.01 [−0.03–0.02]	0.443	0.02 [−0.08–0.11]	0.714
**Water Consumption—Yes vs. No**											
		**iAs%**	***p***	**MMA%**	***p***	**DMA%**	***p***	**PMI**	***p***	**SMI**	***p***
AV area	SMD [95%CI]	−0.72 [−5.30–3.86]	0.755	**6.40 [3.71–9.10]**	<0.001	**−5.68 [−9.89–(–1.46)]**	0.009	**0.40 [0.08–0.71]**	0.015	**−0.77 [−1.17–(−0.37)]**	<0.001
TG area		−2.65 [−8.94–3.64]	0.405	2.81 [−1.43–7.10]	0.192	−0.16 [−6.06–5.74]	0.957	0.18 [−0.13–0.50]SM	0.255	−1.06 [−2.69–0.58]	0.203
Total of 4 areas		−2.06 [−5.80–1.67]	0.277	**5.21 [2.81–7.60]**	<0.001	**−3.15 [−6.62–0.33]**	0.076	**0.33 [0.11–0.55]**	0.003	**−1.07 [−1.85−(–0.30)]**	0.007
**Occupational Exposure to Chemicals—Yes vs. No**											
		**iAs%**	***p***	**MMA%**	***p***	**DMA%**		**PMI**		**SMI**	
AV area	SMD [95%CI]	0.46 [−13.71–14.64]	0.949	−0.32 [−9.35–8.72]	0.945	−0.14 [−13.54–13.26]	0.983	−0.23 [−1.24–0.77]	0.645	0.05 [−1.24–1.35]	0.936
TG area		−3.99 [−10.89–2.91]	0.255	**−8.34 [−12.81–** **(−3.86)]**	<0.001	**12.32 [6.17–18.47]**	<0.001	**−0.32 [−0.66–0.02]**	0.068	**3.64 [1.94–5.34]**	<0.001
Total of 4 areas		−2.00 [−7.83–3.84]	0.501	**−7.65 [−11.40–** **(−3.90)]**	<0.001	**9.65 [4.32–14.98]**	<0.001	**−0.36 [−0.71–(−0.02)]**	0.041	**3.38 [2.22–4.54]**	<0.001
**Smoking—Increment of 1 Pack-Year of Cigarettes**											
		**iAs%**	***p***	**MMA%**	***p***	**DMA%**	***p***	**PMI**	***p***	**SMI**	***p***
AV area	SMD [95%CI]	0.14 [−0.22–0.50]	0.438	0.03 [−0.20–0.26]	0.808	−0.17 [−0.51–0.17]	0.324	−0.00 [−0.03–0.02]	0.800	−0.02 [−0.85–0.02]	0.339
TG area		0.16 [0.16–0.49]	0.323	0.14 [−0.08–0.36]	0.219	**−0.30 [−0.60–0.00]**	0.050	0.00 [−0.01–0.02]	0.803	**−0.07 [−0.15–0.01)]**	0.099
Total of 4 areas		0.16 [−0.77–0.40]	0.183	0.09 [−0.69–0.25]	0.263	**−0.25 [−0.47–** **(−0.03)]**	0.026	0.00 [−0.01–0.01]	0.954	**−0.05 [−0.98–** **0.00]**	0.069
**Wine consumption—Yes (at least 1–2 Glasses Per Day) vs. No**											
		**iAs%**	***p***	**MMA%**	***p***	**DMA%**	***p***	**PMI**	***p***	**SMI**	***p***
AV area	SMD [95%CI]	**6.27 [−1.00–13.54]**	0.090	1.45 [−3.23–6.13]	0.541	**−7.72 [−14.54–(−0.90)]**	0.027	−2.23 [−0.75–0.29]	0.384	**−0.70 [−1.36–(−0.41)]**	0.037
TG area		3.79 [−5.40–12.98]	0.416	0.42 [−5.82–6.66]	0.895	−4.21 [−12.81–4.39]	0.335	−0.22 [−0.68–0.24]	0.344	−0.49 [−2.89–1.92]	0.690
Total of 4 areas		**4.93 [−0.88-10.74]**	0.096	1.11 [−2.75–4.98]	0.570	**−6.04 [−11.45–(−0.64)]**	0.029	−0.21 [−0.56–0.13]	0.226	−0.67 [−1.90–0.57]	0.282
**Alcohol Consumption—Yes (at least 1–2 Glasses Per Day) vs. No**											
		**iAs%**	***p***	**MMA%**	***p***	**DMA%**	***p***	**PMI**	***p***	**SMI**	***p***
AV area	SMD [95%CI]	−2.64 [−16.81–11.53]	0.713	3.75 [−5.25–12.76]	0.411	−1.11 [−14.51–12.28]	0.870	0.16 [−0.85–1.16]	0.757	−0.64 [−1.93–0.65]	0.329
TG area		3.80 [−6.40–14.00]	0.463	2.22 [−4.69–9.13]	0.526	−6.02 [−15.54–3.49]	0.213	−0.03 [−0.55–0.47]	0.893	−0.95 [−3.61–1.72]	0.483
Total of 4 areas		2.66 [−5.41–10.74]	0.516	2.14 [−3.20–7.48]	0.432	−4.80 [−12.33–2.73]	0.210	−0.02 [−0.50–0.46]	0.932	−0.61 [−2.31–1.09]	0.483
**Whole Milk Consumption—Yes (at Least 1–2 Glasses Per Week) vs. No**											
		**iAs%**	***p***	**MMA%**	***p***	**DMA%**	***p***	**PMI**	***p***	**SMI**	***p***
AV area	SMD [95%CI]	2.10 [−2.41–6.61]	0.359	**−2.48 [−5.33–** **0.37]**	0.087	0.38 [−3.89–4.66]	0.859	**−0.32 [−0.64–(−0.01)]**	0.045	0.24 [−0.17–0.65]	0.252
TG area		**6.14 [−0.30–12.58]**	0.062	**−3.39 [−7.77–** **1.00]**	0.129	−2.75 [−8.85–3.34]	0.373	**−0.26 [−0.58–0.06]**	0.114	0.48 [−1.22–2.18]	0.575
Total of 4 areas		2.83 [−0.90–6.55]	0.136	**−2.21 [−4.67–** **0.25]**	0.078	−0.61 [−4.11–2.88]	0.730	**−0.25 [−0.47–(−0.03)]**	0.029	0.07 [−0.72–0.86]	0.868
**Meat Consumption—Yes (at Least 1–2 Times a Week) vs. No**											
		**iAs%**	***p***	**MMA%**	***p***	**DMA%**	***p***	**PMI**	***p***	**SMI**	***p***
AV area	SMD [95%CI]	−1.18 [−5.55–3.19]	0.595	−1.47 [−4.24–1.31]	0.298	2.64 [−1.47–6.75]	0.206	−0.05 [−0.36–0.26]	0.766	0.13 [−0.27–0.53]	0.516
TG area		3.21 [−1.66–8.09]	0.194	**−2.77 [−6.06–** **0.52]**	0.098	−0.45 [−5.04–4.14]	0.848	**−0.28 [−0.52–(−0.04)]**	0.024	0.60 [−0.67–1.88]	0.352
Total of 4 areas		0.36 [−2.85–3.57]	0.826	−1.54 [−3.66–0.57]	0.152	1.19 [−1.81–4.18]	0.437	−0.12 [−0.31–0.07]	0.224	0.11 [−0.57–0.79]	0.746
**Fish Consumption—Yes (at Least 1–2 Times a Week) vs. No**											
		**iAs%**	***p***	**MMA%**	***p***	**DMA%**	***p***	**PMI**	***p***	**SMI**	***p***
AV area	SMD [95%CI]	**5.37 [0.13–** **10.61]**	0.045	0.45 [−2.94–3.84]	0.792	**−5.82 [−10.75–(−0.90)]**	0.021	−0.05 [−0.42–0.33]	0.811	**−0.44 [−0.92–0.04]**	0.073
TG area		1.65 [−4.14–7.44]	0.573	**−3.61 [−7.49–** **0.26]**	0.067	1.96 [−3.46–7.38]	0.475	**−0.24 [−0.52–0.05]**	0.107	0.53 [−0.98–2.04]	0.491
Total of 4 areas		**3.31 [−0.61–7.23]**	0.098	−1.64 [−4.24–0.96]	0.217	−1.67 [−5.35–2.00]	0.371	−0.14 [−0.38–0.09]	0.237	0.04 [−0.79–0.87]	0.921
**Pasta and Cereals Consumption—Yes (at Least 1–2 Times a Week) vs. No**											
		**iAs%**	***p***	**MMA%**	***p***	**DMA%**	***p***	**PMI**	***p***	**SMI**	***p***
AV area	SMD [95%CI]	−1.63 [−26.00–22.73]	0.895	1.51 [−14.00–17.03]	0.847	0.12 [−22.91–23.14]	0.992	0.35 [−1.38–2.08]	0.691	0.122 [−2.10–2.35]	0.913
TG area		5.38 [−14.59–25.34]	0.595	−9.80 [−23.24–3.62]	0.151	4.43 [−14.27–23.13]	0.640	−0.47 [−1.47–0.53]	0.354	1.92 [−3.28–7.12]	0.467
Total of 4 areas		2.55 [−12.68–17.78]	0.742	−5.66 [−15.72–4.39]	0.268	3.11 [−11.11–17.34]	0.667	−0.17 [−1.08–0.74]	0.717	1.14 [−2.06–4.35]	0.483
**AS3MT—Mutated Allele vs. Non Mutated Allele**											
		**iAs%**	***p***	**MMA%**	***p***	**DMA%**	***p***	**PMI**	***p***	**SMI**	***p***
AV area	SMD [95%CI]	−1.47 [−6.22–3.28]	0.542	−1.66 [−4.68–1.35]	0.278	3.13 [−1.33–7.59]	0.168	−0.13 [−0.47–0.20]	0.449	**0.38 [−0.05–0.81]**	0.081
TG area		2.99 [−2.15–8.13]	0.252	−0.95 [−4.45–2.54]	0.591	−2.04 [−6.86–2.79]	0.405	0.02 [−0.24–0.28]	0.866	−0.06 [−1.40–1.29]	0.935
Total of 4 areas		1.03 [−2.48–4.55]	0.563	−1.38 [−3.70–0.94]	0.243	0.34 [−2.94–3.63]	0.836	−0.06 [−0.27–0.15]	0.595	0.20 [−0.54–0.94]	0.599
**GSTT—Wildtype Carrier vs. No Wildtype Carrier**											
		**iAs%**	***p***	**MMA%**	***p***	**DMA%**	***p***	**PMI**	***p***	**SMI**	***p***
AV area	SMD [95%CI]	−3.17 [−8.54–2.20]	0.246	−0.13 [−3.57–3.31]	0.942	3.29 [−1.78–8.37]	0.201	0.06 [−0.33–0.44]	0.773	0.03 [−0.46–0.52]	0.909
TG area		−3.48 [−9.00–2.04]	0.214	**5.35 [1.71–** **9.00]**	0.004	−1.87 [−7.06–3.31]	0.476	**0.25 [−0.03–0.52]**	0.077	−0.75 [−2.19–0.69]	0.303
Total of 4 areas		**−3.56 [−7.42–0.27]**	0.068	**3.10 [0.57–** **5.63]**	0.017	0.47 [−3.14–4.09]	0.797	0.18 [−0.05–0.40]	0.132	0.50 [−1.31–0.32]	0.231

Notes: Bold in table represent statistically significant results.
